# Spectral Properties of Complex Distributed Intelligence Systems Coupled with an Environment

**DOI:** 10.3390/e27101016

**Published:** 2025-09-27

**Authors:** Alexander P. Alodjants, Dmitriy V. Tsarev, Petr V. Zakharenko, Andrei Yu. Khrennikov

**Affiliations:** 1 National Center for Cognitive Reaserch, ITMO University, St. Petersburg 197101, Russia; alexander_ap@list.ru (A.P.A.); dmitriy_93@mail.ru (D.V.T.); p.zaxarencko2015@yandex.ru (P.V.Z.); 2 International Center for Mathematical Modeling in Physics, Engineering, Economics, and Cognitive Science Linnaeus University, S-35195 Vaxjo-Kalmar, Sweden

**Keywords:** open complex networks, distributed intelligent systems, renormalization group, spectral entropy, phase synchronization, LLM

## Abstract

The increasing integration of artificial intelligence agents (AIAs) based on large language models (LLMs) is transforming many spheres of society. These agents act as human assistants, forming Distributed Intelligent Systems (DISs) and engaging in opinion formation, consensus-building, and collective decision-making. However, complex DIS network topologies introduce significant uncertainty into these processes. We propose a quantum-inspired graph signal processing framework to model collective behavior in a DIS interacting with an external environment represented by an influence matrix (IM). System topology is captured using scale-free and Watts–Strogatz graphs. Two contrasting interaction regimes are considered. In the first case, the internal structure fully aligns with the external influence, as expressed by the commutativity between the adjacency matrix and the IM. Here, a renormalization-group-based scaling approach reveals minimal reservoir influence, characterized by full phase synchronization and coherent dynamics. In the second case, the IM includes heterogeneous negative (antagonistic) couplings that do not commute with the network, producing partial or complete spectral disorder. This disrupts phase coherence and may fragment opinions, except for the dominant collective (Perron) mode, which remains robust. Spectral entropy quantifies disorder and external influence. The proposed framework offers insights into designing LLM-participated DISs that can maintain coherence under environmental perturbations.

## 1. Introduction

Rapid advancement of artificial intelligence (AI) models—particularly large language models (LLMs)—has positioned them as a central component in decision support and information exchange within Distributed Intelligence Systems (DISs) [1]. DISs can be conceptualized as multi-agent systems composed of artificial intelligence agents (AIAs), typically embodied as digital assistants (“avatars”), and their users, natural intelligence agents (NIAs), namely, humans [2]. Among AIAs, LLMs are gaining widespread adoption [3,4,5]. However, enhancing the quality of human–LLM interaction challenges the broader development of AI [5,6].

AI agents in DISs can perform various tasks autonomously or can collaborate as teammates working along with humans and other AI agents [7]. Due to ongoing information exchange, DISs can self-organize into groups that develop shared opinions and collective preferences concerning the topics under discussion.

Physically, a DIS represents an open system structured as a network facilitating interactions between NIAs and AIAs, as well as with external information sources accessible to both. As a consequence, even under relatively simple interaction rules, the behavior of DISs can become highly complex and difficult to predict [8,9]. The topology of the underlying DIS network plays a crucial role in shaping the system dynamics [5,10]. In general, a DIS can be modeled as a complex network containing hubs—nodes that function as centers of influence in decision-making (DM), cf. [11]. In this context, investigating the complexity of interactions between NIAs and AIAs within DISs and exploring ways to reduce this complexity becomes a practically motivated fundamental task. This task is imperative to understand the formation and dissemination of collective opinions, which are independent of external (to DIS) influences, as well as to facilitate collective decision-making, where possible, cf. [12,13].

It should be noted that the study of social effects, DM processes, and formation of opinions in multi-agent systems, which takes into account interactions between humans and AI agents based on LLMs, has recently received a great deal of attention, see, for example [14,15,16,17]. For example, Zhang et al., review human-AI collaborative DM tasks, highlighting the key categories of such problems, types of AI support, and evaluation metrics [15]. In [16], the authors propose a novel approach to modeling opinion dynamics using networks of LLM-based agents. They identify properties of these agents that enable them to reach consensus. Of particular interest to us is the context of studying social influence as a reservoir for collective DM are the results presented in [17]. The authors examine the issue of influence (social pressure) exerted by a group of AI agents (which can be considered a reservoir) on their users, effectively prompting a change of opinion.

Despite the variety of practical human–AI interaction tasks, the long-studied problems of opinion formation and social dynamics are clearly evident at the core; see, for example, [18,19,20]. In this regard, we would like to highlight our work [21], in which we use a laser-like model to identify the effects of large enough social amplification and formation of echo chambers (analogous to laser resonators) within an open system of binary-DM agents. It should be emphasized that neither our initial study [21] nor our subsequent work [22] investigates the impact of agents’ network environment on opinion formation within the target community.

In this paper, we examine the formation of opinions and decisions within a DIS actively interacting with its environment, modeled as an incoherent information reservoir. We study the phase synchronization between the DIS agents and the applicability of the renormalization group approach in two limiting cases: of network–reservoir interaction: (i) commuting adjacency and coupling with reservoir matrices, and (ii) non-commuting matrices. The study of these issues has an important practical significance in sociodynamics. In particular, we assume that the external environment relative to the target community represents a certain external (graph-based) filter capable of “cleansing” the set of opinions, taking into account the structure of social connections, cf. [23]. In this case, (i) corresponds to the limit where the filter weights are fully aligned with the network topology. In the second case (ii), such an alignment is absent, which ultimately affects the cooperation of DM agents (cf. [24]).

The structure of the article is as follows. In Section 2, we briefly discuss works that help the reader assess the relevance and significance of our work within the context of ongoing research worldwide. Section 3 provides a general theoretical framework for our study, outlining the methodologies and approaches employed. Particular attention is paid to the network architecture of the DIS and the conditions governing its interaction with the external environment (reservoir). In this context, we employ the Gorini–Kossakowski–Sudarshan–Lindblad (GKSL) master equation for the density operator $\hat{\rho }$, which allows accounting for various driven-dissipative processes within the DIS network. Section 4 presents the core results of our work. We analyze characteristic patterns in the spectral properties of the DIS network, focusing on their implications for information dissemination. This analysis is carried out for two limiting cases of interaction between the reservoir and DIS network: (i) when the adjacency matrix commutes with the matrix governing the reservoir, and (ii) when the matrices do not commute. We identify the renormalization group (RG) criteria under which the network properties can be scaled to facilitate efficient information transmission. A key aspect of our analysis involves the entropy of the eigenvalue spectrum, which provides insight into distinct propagation modes. In Section 5, we address the synchronization problem in DIS networks. Here, we investigate the conditions under which such networks exhibit synchronizability when coupled to an external environment. Finally, in the Conclusion, we summarize our main findings and discuss their implications, outlining future research.

## 2. Related Works

Our quantum-like method of studying information propagation processes in DIS, formulated in a fairly general problem setting, can be interpreted as a Quantum-like Graph Signal Processing (QGSP) approach. It generalizes the currently relevant classical Graph Signal Processing (GSP) approach, in which data is defined on the nodes of a graph, see e.g., [23,25,26]. For this reason, the applicability of GSP methods and approaches has a great potential. The applications include Graph Neural Networks (GNNs) [27], neuroscience (brain signal processing) [28], image processing [29], and sensor networks and the Internet of Things (IoT) [30]. Graph spectral filters and transforms allow both local and global relationships between objects to be modeled, enabling more accurate processing of structured data in such tasks as classification, regression, clustering, recommendation systems, and semantic analysis. Classical GSP is widely used in sociodynamics, where a social system is represented as a graph of interactions and where opinions, preferences, or levels of trust are treated as signals on this graph. By applying GSP tools, one can formalize and analyze information diffusion, consensus formation, belief dynamics, and influence propagation in social networks. This is particularly important when studying multidimensional and thematically interconnected discussions, as it is necessary to consider both structural and contextual dependencies between topics [23,25].

The fundamental differences between the QGSP and classical GSP approaches can be understood by comparing classical and quantum walks on graphs. Classical random walks on graphs are modeled as stochastic Markov processes, where transitions from one vertex to another depend solely on the current state and transition probabilities [31]. In contrast, quantum walks on graphs describe the evolution of the system state via a unitary transformation that captures the interference effects of all possible paths through the graph, cf. [32].

This work focuses on the spectral properties of DIS networks that represent open (thermodynamically non-equilibrium) systems interacting with their environment (reservoir). Our analysis demonstrates that these spectral properties are strongly influenced by the nature of the DIS-environment interaction. This aspect underlies the practical relevance and significance of our study, although the spectral properties of complex networks have been extensively studied in the literature (see, e.g., [33,34,35,36,37,38,39]). Specifically, this article focuses on two important issues.

First, we study how information disseminates in DIS networks where coherent and incoherent processes co-occur. Quantum theory provides an effective mathematical framework for studying complex open systems, such as lattices and networks, cf. [40,41,42]. The framework enables the analysis of the measurement effect [43,44], and robustness of these systems during information distribution or processing in the presence of disorder [45].

Coherent effects arise from the interference of various possible decision paths, which can either enhance or suppress the spread of information in social systems [46,47]. These effects are often modeled using quantum-like frameworks, where decisions are made under uncertainty and associated with interference between multiple potential implementations [48,49]. In parallel, we also consider incoherent processes related to classical diffusion mechanisms in networks, cf. [50,51,52]. To model these combined processes comprehensively, we adopt a quantum probability approach. In particular, we employ the GKSL master equation, which accounts for system–reservoir interactions and is well suited to modeling dissipative dynamics in various systems, cf. [53,54,55,56].

Second, we investigate under what conditions the topology of a complex network can be effectively simplified (convoluted) in the context of information propagation. This problem is of significant interest in both classical and quantum information science, cf. [57,58]. This simplification involves merging a subset of nodes or edges according to specific rules, without altering the network’s statistical or functional properties. This process is analogous to the renormalization group (RG) approach in quantum physics [59], originally developed to describe spin-1/2 particles on a two-dimensional lattice in the Ising model. Today, RG methods are widely used to reduce the complexity of various network systems, including neural networks, and have become powerful tools to accelerate machine learning tasks [60].

In our recent work [61], we demonstrated that the RG behavior emerges in open, driven-dissipative network structures. In the present study, we explore the characteristics of external reservoirs that caused violations of the RG hypothesis.

**Figure 1:**
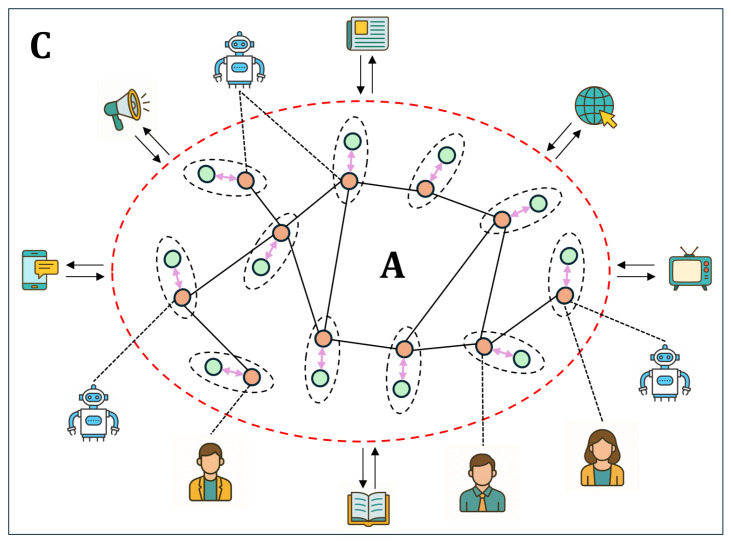
Sketch of the DIS network (system A) interacting with the environment (system C). The nodes of the DIS represent pairs consisting of an AIA (light brown circles) and an NIA (green circles). The system C serves as a large external information reservoir that includes mass media, external NIAs, AIAs, and other sources of information.

As a part of previous studies, we proposed a DIS consisting of pairs of users and their digital assistants (avatars), connected by an avatar–avatar complex graph (see Figure 1). The DIS aimed to maximize average user satisfaction resulting from AIA–NIA interaction. The results obtained demonstrate the DIS complex behavior due to the network topology. Moreover, ensuring the long-term viability of digital assistants in the multi-agent systems under consideration was identified as an important aspect of adapting them to users (see [8]). In [21,22], we employed a quantum probability approach to examine the complicated behavior of DM agents in a DIS. It was shown that AIAs tend to self-organize under certain conditions specified by the properties of NIAs. The advantage of a quantum description for complex systems is that it can take into account both incoherent and coherent processes, when spreading information within these systems. In this sense, such systems exhibit properties similar to those of physical lasers based on disordered and gain systems, which can amplify information at certain frequencies, cf. [62,63,64]. However, in [21,22], we did not consider the specifics of the complex DIS network that emerges when interacting with the environment. Specifically, we assumed that such a network could coherently distribute information within the DIS. The present work focuses on studying information dissemination in complex networks representing open systems, i.e., systems interacting with the environment.

## 3. Theoretical Background

In this section, we present a general framework of our study, outlining the methods and quantum-inspired approaches employed. The primary focus is on formulating the problem of how the information reservoir influences the DIS, which is a targeted community of decision-making agents.

### 3.1. Dis Network Models

In this study, we consider a model composed of the DIS network (target system A) and its environment (system C), illustrated in Figure 1. Each node in the network hosts a pair consisting of an AIA and NIA, which are considered to form a robust unit. The AIAs—such as avatars, digital twins, and other autonomous systems—are interconnected via an AIA–AIA complex network that enables direct information exchange among them by means of social information quanta—s-photons, which represent socially meaningful text messages, cf. [21,47]. In contrast, NIAs do not communicate directly with one another, but can receive information indirectly through interactions with the external environment. AIAs also have access to external information, making the DIS fully open. The environment (denoted as system C in Figure 1) serves as an information reservoir, employing various facilities of interaction with agents of the target system A. In particular, we can recognize the reservoir as a network external to the DIS possessing a defined topology and weighted connections with the DIS. In this regard, the present work aims to investigate how the configuration of this reservoir affects the emergence of a spectrum of opinions, moods, and other forms of shared cognition, cf. [65].

**Figure 2:**
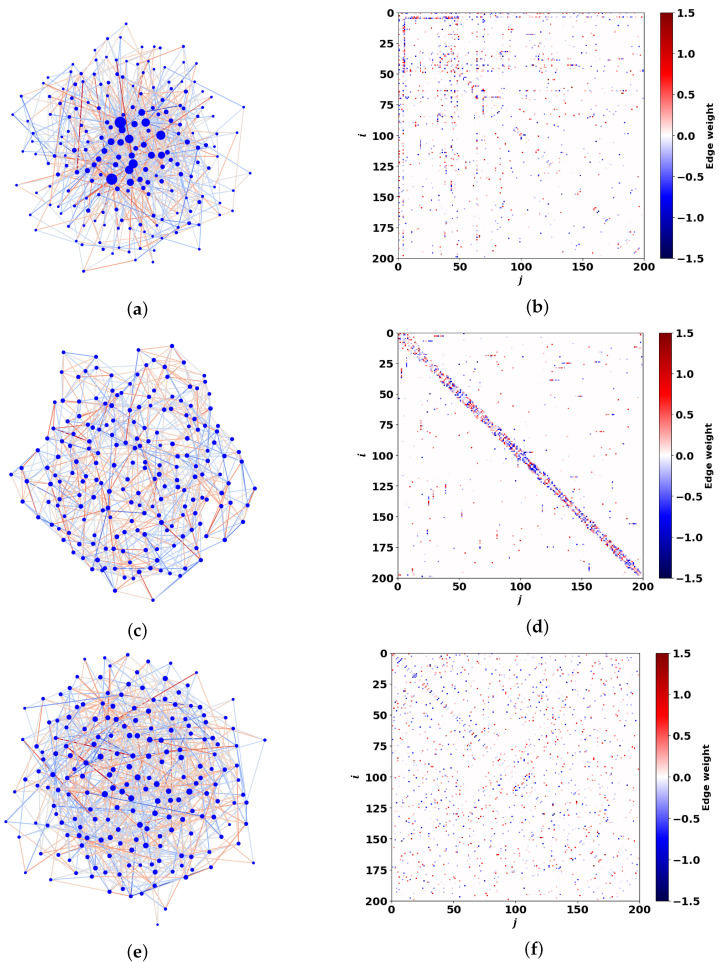
(**a**) Scale-free and Watts–Strogatz (**c**,**e**) weighted connectivity graphs specify the coupling of the DIS network, comprising N=200 nodes, with the external reservoir. The parameters are: degree exponent ν=2.5 for (**a**), and β=0.1, β=0.9 for (**c**), (**e**), respectively. Graphs (**b**), (**d**), (**f**) relate to connectivity matrices $\hat{C}$ for the graphs given in (**a**), (**c**), (**e**), respectively. Matrix elements $C_{ij}$ are normally distributed with mean value ${\bar{C}}_{ij}=0$ and standard deviation σ=0.5. See other details in the text.

Let us consider the DIS network models that we analyze in this work. Figure 2 shows these network graphs, which are specified by a square N×N adjacency matrix $\hat{A}=\left(A_{ij}\right)$, i,j=1,…,N. We assume that each AIA–NIA pair occupies one of the *N* nodes in the DIS network, see Figure 1. We analyze two important cases for the matrix elements of the adjacency matrix $\hat{A}$. First, we treat the elements of matrix $\hat{A}$ as random binary variables, taking values 0 and 1 for *unweighted* graphs, respectively. Specifically, $A_{ij}=1$ if two arbitrary nodes in the graph are connected by an edge, and $A_{ij}=0$ if they are not. Since we do not consider loops for graph nodes, diagonal matrix elements $A_{ii}=0$, cf. [66].

Secondly, one of our approaches within this work uses *weighted* graphs for characterization of a DIS network reservoir. The approach implies inhomogeneous coupling between the DIS network and the reservoir, described by matrix $\hat{C}$, which is constructed as follows:

(1) $$\left\{\begin{array}{c} C_{ij}=0,\;\;\;\;\;\;\;\;\;\;\;\;\;\;\;\;\;if\;A_{ij}=0;\\ C_{ij}∼N\left({\bar{C}}_{ij},\sigma^{2}\right),\;\;if\;A_{ij}=1. \end{array}\right.$$

Since we consider undirected graphs, matrices $\hat{A}$ and $\hat{C}$ are symmetric. In this work, $\hat{C}$ presents the connectivity matrix that couples a DIS with the external reservoir. $\hat{C}$ repeats the DIS agents’ connections established by $\hat{A}$. In particular, in (1), for each nonzero element $A_{ij}$ of the adjacency matrix, a corresponding random coupling strength, $C_{ij}=C_{ji}$, is assigned, drawn from a normal (Gaussian) distribution with mean ${\bar{C}}_{ij}$ and standard deviation σ. From a practical point of view, (1) means that agents outside the DIS forming the reservoir are involved in all communications between DIS agents, but with different weights. In this sense, we can recognize matrix $\hat{C}$ as the influence matrix. The latter plays an essential role in the study of complex systems involving different subsystems and an external bath, cf. [19,67,68].

The statistical properties of the graph are evaluated by first (〈k〉) and normalized second (ζ) moments of the degree distribution. These are defined as follows:

(2a) $$\begin{array}{c} \begin{array}{c} \langle k\rangle =\frac{1}{N}\sum_{i=1}^{N}k_{i}=\frac{1}{N}\sum_{i=1}^{N}\sum_{j=1}^{N}A_{ij}; \end{array} \end{array}$$

(2b) $$\begin{array}{c} \begin{array}{c} \zeta \equiv \frac{\langle k^{2}\rangle }{\langle k\rangle }=\frac{1}{N\langle k\rangle }\sum_{j=1}^{N}k_{j}^{2}, \end{array} \end{array}$$

where $k_{j}$ determines the *j*-th node connectivity.

Figure 2 presents *weighted* scale-free and Watts-Strogatz (WS) graphs constructed to model connectivity $\hat{C}$ between the DIS and the reservoir. As an illustrative example, here we assume that the matrices in Figure 2 obey condition (1). Notably, Figure 2 also presents the DIS network topology, albeit without weights. The graphs in Figure 2 were generated by the igraph library of the R language and then processed via Python 3.0 NetworkX. For the scale-free graph in Figure 2a, the procedure is established by a simple *N*-step loop, which adds a node and ensures that the graph fulfills a power-law distribution of node degrees in each step. Figure 2b displays the connectivity matrix of the weighted scale-free graph shown in Figure 2a. In particular, the DIS network in this case possesses 〈k〉=7.8 and a number of hubs, the largest of which has node degree $k_{max}=71$, i.e., it is connected to the majority of other nodes. This issue is also reflected in the large normalized second moment ζ=19. For large *N*, one can approximate a scale-free network by a continuous power-law degree distribution (PLDD), w(k), in the form

(3) $$\begin{array}{c} w\left(k\right)=\frac{\left(\nu -1\right)k_{min}^{\nu -1}}{k^{\nu }}, \end{array}$$

where ν is the degree exponent, $k_{min}$ is the smallest node degree; for a scale-free graph in Figure 2a, the degree exponent is ν=2.4.

The WS graph allows examination of the transition from a regular (β=0) to a random (β=1) graph by using rewiring probability β∈[0,1]; cf. [69]. In particular, at β=0 WS graph represents a regular graph with node degrees $k_{i}=\langle k\rangle =\langle k^{2}\rangle$, which gives the normalized second moment, $\zeta =\langle k^{2}\rangle /\langle k\rangle =\langle k\rangle$. At β=1, the WS graph becomes random with a Poisson distribution

(4) $$\begin{array}{c} w\left(k\right)=\frac{{\langle k\rangle }^{k}e^{-\langle k\rangle }}{k!}, \end{array}$$

with the same 〈k〉 and ζ=〈k〉+1.

The WS graphs in Figure 2c,e were generated in two steps. First, a regular graph is designed: each of the *N* nodes is connected with 8 closest neighbors, which gives $k_{i}=8$ for all *i* and thus 〈k〉=8. Then, each of the links is rewired with probability β, ensuring that no loops are generated. During the second step, the average node degree is maintained with the maximal and minimal degrees as well as the second normalized moment, ζ, changing.

Figure 2c,e demonstrate the WS graphs plotted at β=0.1 and β=0.9, respectively. The WS graphs demonstrate no sufficient hubs; the maximal node degrees are $k_{max}=11$ and $k_{max}=16$ for graphs in Figure 2c,e. These graphs possess normalized second moments ζ=8.2 and ζ=8.9, respectively; they are close to their average degrees 〈k〉=8.

### 3.2. Basic Equations

Let us consider the network model represented by AIA-AIA connection graphs in Figure 2. The Hamiltonian of the multiagent network system can be represented in the form

(5) $$\begin{array}{c} \begin{array}{c} {\hat{H}}_{I}=-\frac{J_{R}}{2}\sum_{i,j=1}^{N}A_{ij}\left({\hat{a}}_{i}^{†}{\hat{a}}_{j}+{\hat{a}}_{j}^{†}{\hat{a}}_{i}\right), \end{array} \end{array}$$

where ${\hat{a}}_{i}^{†},{\hat{a}}_{i}$ are the s-photon creation and annihilation operators, respectively; $J_{R}>0$ is a real-defined AIA-AIA coupling strength; and $A_{ij}$ are the symmetric network adjacency matrix $\hat{A}$ elements.

We can account for the driven-dissipative properties by exploring the GKSL master equation for density operator $\hat{\rho }$, that is (cf. [61])

(6) $$\begin{array}{c} \dot{\hat{\rho }}=-i\left[{\hat{H}}_{I},\hat{\rho }\right]+\sum_{i}\kappa_{i}\left(2{\hat{a}}_{i}\hat{\rho }{\hat{a}}_{i}^{†}-{\left\{{\hat{a}}_{i}^{†}{\hat{a}}_{i},\hat{\rho }\right\}}_{+}\right)+\frac{J_{I}}{2}\sum_{i,j}C_{ij}\left(2\hat{Z}\hat{\rho }{\hat{Z}}^{†}-{\left\{{\hat{Z}}^{†}\hat{Z},\hat{\rho }\right\}}_{+}\right)+\\ +\sum_{i}p_{i}\left(2{\hat{a}}_{i}^{†}\hat{\rho }{\hat{a}}_{i}-{\left\{{\hat{a}}_{i}{\hat{a}}_{i}^{†},\hat{\rho }\right\}}_{+}\right), \end{array}$$

where the dot over $\hat{\rho }$ denotes to derivative with respect to time; $\left[\hat{A},\hat{B}\right]=\hat{A}\hat{B}-\hat{B}\hat{A}$ and ${\left\{\hat{A},\hat{B}\right\}}_{+}=\hat{A}\hat{B}+\hat{B}\hat{A}$ are the commutator and anti-commutator, respectively.

In (6), the first term characterizes coherent information exchange between the *i*-th and *j*-th AIAs, while the last three terms include their interaction with the huge external information reservoir. In particular, the second term in (6) with $\kappa_{i}$ characterizes the information leakage rate in the *i*-th AIA. The third term in (6) specifies incoherent information transfer between the *i*-th and *j*-th AIAs with rate $C_{ij}$. Thus, we can recognize $\hat{C}$ as the matrix of connections of the DIS network with the reservoir. Since matrix elements $C_{ij}$ take random values, Equation (6) is therefore a GKSL equation with random coefficients.

In (6), we also introduce the jump operator $\hat{Z}={\hat{a}}_{i}+e^{i\theta }{\hat{a}}_{j}$ that specifies the interaction of AIAs with reservoir determined by the $\hat{Z}$ operator and relative phase θ; θ specifies the phase shift induced by the reservoir with respect to *i* to *j*, or *j* to *i* couplings, cf. [54,70]. In (6), the term with $p_{i}$ defines the rate of information field injection in the *i*-th AIA, as shown in Figure 1.

Below, we are interested in the mean-field approximation. From (5) and (6) for average information field $E_{i}=\langle {\hat{a}}_{i}\rangle$ of the *i*-th AIA, we obtain the rate

(7) $${\dot{E}}_{i}=-\left(K_{i}-p_{i}\right)E_{i}+iJ_{R}\sum_{j=1}^{N}A_{ij}E_{j}-\cos\theta J_{I}\sum_{j=1}^{N}C_{ij}E_{j},\;\;\;\;\;\;i=1,\ldots ,N,$$

where we introduce effective information loss parameter $K_{i}=\kappa_{i}+J_{I}\sum_{j}C_{ij}$ for the *i*-th AIA that accounts for the influence of network tunneling losses and the node weighted connectivity. Notice that for unweighted matrix elements $C_{ij}$ for incoherent reservoir interactions $J_{I}\sum_{j}C_{ij}\to J_{I}\sum_{j}A_{ij}=k_{i}J_{I}$, where $k_{i}$ is the *i*-th node degree, cf. [61]. In a more general case of weighted adjacency matrix elements $C_{ij}$, this term cannot be reduced to $k_{i}$.

We examine stationary states $E_{i}=E_{i}^{\left(n\right)}e^{-i\omega_{n}t}$ for (7), where $\omega_{n}$ is a set of eigenfrequencies for the network of a DIS. In this case, from (7) we obtain

(8) $$\hat{M}{\vec{E}}^{\left(n\right)}=\omega_{n}{\vec{E}}^{\left(n\right)},$$

where ${\vec{E}}^{\left(n\right)}={\left(E_{1}^{\left(n\right)},\ldots ,E_{N}^{\left(n\right)}\right)}^{T}$ is an *N*-component eigenvector corresponding to the *n*-th eigenvalue $\omega_{n}$ for the matrix $\hat{M}$ defined as

(9) $$\hat{M}=-J_{R}\hat{A}-i\left(\left(K_{i}-p_{i}\right)\hat{I}+\cos\theta J_{I}\hat{C}\right),$$

where $\hat{I}$ is the identity matrix. From a practical point of view, the set ${\left\{\omega_{n}\right\}}_{n=1}^{N}$ of eigenfrequencies determines the variability or spectrum of opinions formed in the DIS as a result of information exchange.

Noteworthy, in (9), matrix $\hat{M}$ defines the combined system of the DIS network and its reservoir in a semiclassical limit (within mean-field theory) that does not account for quantum correlations between DIS agents and the information field (see Figure 1). The first term in (9) defines coherent processes of information distribution within the network, while the second term in (9) characterizes information diffusion in the presence of AIA and NIA interactions with the reservoir. Thus, the variability of opinions formed in the DIS is completely determined by the spectrum of the $\hat{M}$ matrix, which is random, non-Hermitian, and sparse, cf. [71]. For further analysis, it is useful to specify the nature of matrix $\hat{M}$.

We assume that the lack of information in the DIS (parameter $K_{i}$) can be compensated by strong enough external information pumping $p_{i}$ for any *i*-th node. Thus, our further analysis relates to matrix $\hat{M}$ given in the form (cf. (9))

(10) $$\hat{M}=-J_{R}\hat{A}-i\cos\theta J_{I}\hat{C}.$$

Note that matrix $\hat{M}$ in (10) does not contain any diagonal elements. Within the framework of opinion and consensus formation problems this situation can be associated with the limit at which the self-confidence of agents can be neglected, cf. [72,73]. In other words, agents are completely influenced by external conditions. This situation may be assumed to occur in a significantly non-equilibrium social system, such as a social laser [21].

We then examine two limiting cases of matrix $\hat{M}$, which arise from the algebraic properties of matrices $\hat{A}$ and $\hat{C}$.

First, we examine the case when $\hat{A}$ and $\hat{C}$ commute with each other, i.e.,

(11) $$[\hat{A},\hat{C}]=0.$$

Equation (11) implies that matrix $\hat{C}$ is either a function of $\hat{A}$ in its basis or it belongs to the algebra generated by $\hat{A}$. In this case, matrices $\hat{A}$, $\hat{C}$, and $\hat{M}$ are simultaneously diagonalizable in the same basis. Consequently, the frequency eigenvalues of these matrices satisfy the following relation:

(12) $$\omega_{M,n}=-J_{R}\omega_{A,n}-i\cos\theta J_{I}\omega_{C,n}.$$

As a result, we can represent spectral density $\rho_{M}\left(\omega \right)$ in the form

(13) $$\rho_{M}\left(\omega \right)=\int \int \delta \left(\omega +J_{R}\omega_{A}+i\cos\theta J_{I}\omega_{C}\right)\rho_{AC}\left(\omega_{A},\omega_{C}\right)d\omega_{A}d\omega_{C},$$

where $\rho_{AC}\left(\omega_{A},\omega_{C}\right)$ is the joint spectral measure (or spectral density) of two commuting diagonalizable matrices $\hat{A}$ and $\hat{C}$. It describes the joint distribution of pairs of their eigenvalues. In (13), we moved to continuous frequency variables.

Condition (11) obviously holds if matrix $\hat{C}$ can be represented as

(14) $$\hat{C}=\sum_{m=1}^{K}\alpha_{m}{\hat{A}}^{m},$$

where at least one of $\alpha_{m}$ is non-zero; $K\in Z_{>0}$. In the theory of GSP (14), there is nothing else as an expression for the dependence of a polynomial filter based on the adjacency matrix, [74], cf. [23,26]. In this case, *K* specifies the order of the polynomial filter and limits the maximum length of paths considered during filtering. Note that there is also a generalization of classical GSP approaches to quantum theory [75].

From a practical point of view, the K>1 case describes how the information reservoir affects DIS agents indirectly, via their connections in the network. As a practically important illustrative choice of matrix $\hat{C}$, in this work, we assume that

(15) $$\hat{C}=\hat{A}.$$

Equation (15) shows that the information reservoir directly affects the agent.

Equation (15) allows the introduction of the complex coupling parameter for the AIA-AIA network in the form

(16) $$\begin{array}{c} \begin{array}{c} J=J_{R}+i\cos\theta J_{I}\equiv J_{R}+iJ_{I,eff}\equiv \left|J\right|e^{i\Theta }, \end{array} \end{array}$$

where Θ angle is specified later. Equation (16) allows us to recast $\hat{M}$ matrix as

(17) $$\begin{array}{c} \begin{array}{c} \hat{M}=-J\hat{A}. \end{array} \end{array}$$

Thus, here we suppose that the interaction with the reservoir reduces to a homogeneous stile, i.e., node-independent, incoherent coupling between AIAs that repeats properties of adjacency matrix $\hat{A}$ with scaling factor −J.

Second, we assume that interaction with the reservoir is essentially *inhomogeneous* and reduces to *node-dependent* incoherent coupling strength between AIAs. In this limit, we establish $\hat{C}$ as a random matrix that possesses properties specified in (1). Notably, in this case, matrices $\hat{A}$ and $\hat{C}$ do not commute, and relation (12) for their spectral properties is not valid. In this case, the results presented below have a natural interpretation within quantum theory: non-commuting operators associated with observable quantities cannot possess a common eigenstate, see e.g., [76].

## 4. Results

In this section, we present the main findings of our work. The focus is on the spectral properties of matrix $\hat{M}$, which incorporates both the coherent contribution of the adjacency matrix, capturing the internal connections among agents, and the generally incoherent influence exerted by the reservoir.

### 4.1. Renormalization Group for Field Eigenstates

In general, the spectral characteristics of matrix $\hat{M}$ are sufficiently complicated for theoretical analysis as the DIS agents interact with an external information reservoir in a complex and sometimes unpredictable way. However, we can consider the limiting cases (see (9)) of interaction in the DIS, when the spectrum exhibits features that allow simplifying the analysis of information distribution in the network. We start from the first case described by (15) and (16). In this limit, from (8) we obtain

(18) $$\omega_{n}E_{i}^{\left(n\right)}+J{\bar{E}}_{A,i}^{\left(n\right)}=0,\;\;\;\;\;i=1,\ldots ,N.$$

where we introduce the *local* average information field that *coherently* acts on the *i*-th node from its neighbors as (cf. [61])

(19) $${\bar{E}}_{A,i}^{\left(n\right)}=\sum_{j=1}^{N}A_{ij}E_{j}^{\left(n\right)}=\left|{\bar{E}}_{i}^{\left(n\right)}\right|e^{i\chi_{A,i}^{\left(n\right)}},$$

where $\chi_{A,i}^{\left(n\right)}$ is the phase of the local average field.

For that, we can write

(20) $${\bar{E}}_{A,i}^{\left(n\right)}=\eta_{i}^{\left(n\right)}E_{i}^{\left(n\right)},$$

where $\eta_{i}^{\left(n\right)}=\left|\eta_{i}^{\left(n\right)}\right|e^{i\chi_{A,i}^{\left(n\right)}}$ is a *scaling parameter* that defines the amplitude (strength) with which the local field of the *n*-th mode affects the *i*-th AIA. The RG condition states that

(21) $$\eta_{i}^{\left(n\right)}=\eta^{\left(n\right)}$$

is *real* and the same for a given *n* and all *i*.

The annealing network approach allows finding a simple solution to Equation (18) that uses representation of adjacency matrix elements in the form (cf. [77])

(22) $$A_{ij}=\frac{k_{i}k_{j}}{N\langle k\rangle }.$$

Inserting (22) into Equation (18), we obtain

(23) $$\omega_{n}E_{i}^{\left(n\right)}+Jk_{i}\bar{E}=0,$$

where we define the *global* average information field within DIS as

(24) $$\bar{E}=\frac{1}{N\langle k\rangle }\sum_{j=1}^{N}k_{j}E_{i}^{\left(n\right)}.$$

Multiplying (23) by $\frac{1}{N\langle k\rangle }\sum_{i=1}^{N}k_{i}$ and using (2b) and (24), we obtain an expression for frequency $\omega_{n}$ in the form

(25) $$\omega_{P}\equiv \omega_{n}=-J\zeta =-J_{R}\zeta -iJ_{I,eff}\zeta .$$

Equation (25) is important from a practical point of view. The real part of frequency ω describes the coherent, periodic evolution of the information field in DIS. In this case, the imaginary part of the frequency in (25) specifies amplification (if $J_{I,eff}<0$) or attenuation (if $J_{I,eff}>0$) of average information field $\bar{E}$. Our analysis and numerical estimations presented below show that ω is close to the Perron eigenfrequency evaluation. Combining Equation (25) with (18) and (20), we obtain the RG scaling factor for Perron mode $\eta_{i}^{\left(P\right)}$ in this limit as

(26) $$\eta_{i}^{\left(P\right)}=\zeta .$$

**Figure 3:**
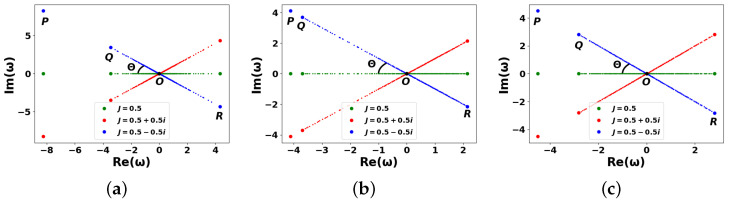
Dimensionless imaginary parts Im(ωn) vs. the real ones, Re(ωn), for eigenfrequencies $\omega_{n}$ for DISs described by graphs in Figure 2 at different complex tunneling rates $J=J_{R}+iJ_{I,eff}$ for: (**a**) scale-free, (**b**,**c**) WS graphs calculated at β=0.1 (**b**), and β=0.9 (**c**), respectively. The other parameters are N=200, $J_{R}=0.5$; Θ is specified in (27). See more details in the text.

Figure 3 presents the numerical solution of Equation (18), illustrating the dependence of the imaginary part of the eigenfrequencies $\omega_{n}$ on their real part for a dissipative scale-free (Figure 3a) and WS (Figure 3b,c) DIS networks, respectively. In particular, Figure 3 shows the results obtained from a single realization of the $\hat{M}$ matrix. Multiple simulations with random realizations of the $\hat{M}$ matrix were also performed and averaged to obtain the dependencies in Figure 3. However, no statistically significant trends or regularities were observed.

Figure 3 demonstrates several remarkable points—*O*, *P*, *Q*, and *R*, which reflect distinct spectral features of the system. A key aspect of the spectrum is the emergence of an isolated Perron eigenvalue (PE)—the eigenvalue with the largest absolute value indicated by the leftmost points *P* in Figure 3; see also (25). This phenomenon is consistent with the Perron–Frobenius theorem and arises from the spectral properties of the adjacency matrix of complex networks. In particular, the PE for a scale-free graph is known to reside the modulus of its real part, |Re(ωP)|, within the interval $\left[\langle k\rangle ,k_{max}\right]$, cf. [35,37]. The second and third largest eigenvalues following the PE are denoted by points *Q* and *R* in Figure 3: the *R* points correspond to the rightmost modes in the complex plane, while the *Q* points are associated with intermediate spectral features. Point *O* represents the central region of the spectrum, characterized by eigenfrequencies satisfying $\omega_{O}≃0$. Relevant modes are typically associated with resonant or long-lived dynamics in the network.

At $J_{I}=0$, the networks in Figure 3 provide only coherent information transfer between the nodes. In this limit, all eigenfrequencies $\omega_{n}$ occupy the line Im(ω)=0; see the green lines in Figure 3.

When $J_{I}\neq 0$, the system enters a regime where both AIAs and NIAs interact with an external information reservoir. On the contrary, if $J_{R}=0$, the DIS network becomes purely driven-dissipative, characterized by eigenfrequencies aligned vertically in the complex plane (not shown in Figure 3). In the general case, when $J_{I},J_{R}\neq 0$, the eigenfrequencies $\omega_{n}$ are distributed along the straight lines inclined at angle Θ with respect to the Im(ω)=0 axis. Notably, the modes located in domain Im(ω)>0 are amplified, whereas those with Im(ω)<0 are rapidly damped. For instance, when θ=0 (see the red lines in Figure 3, corresponding to Θ>0), eigenmodes with Re(ωn)>0 experience amplification, while those with Re(ωn)<0 are suppressed. Conversely, the blue lines (associated with θ=π, where Θ<0) exhibit the opposite behavior: the modes with Re(ωn)<0 are amplified, while those with Re(ωn)>0 are damped.

Angle Θ can be estimated using simple geometric considerations applied to specific spectral points in Figure 3. In particular, one can see that for an arbitrary mode, *n*, the angle, Θ, approaches (cf. [61])

(27) tan(Θ)=Im(ωn)Re(ωn)=JI,effJR

Figure 3 exhibits the fulfillment of RG conditions (19)–(21). Geometrically RG conditions appear as a result of simple affine transformation (scaling) of matrix $\hat{A}$ eigenvalue $\omega_{n}\to -J\omega_{n}$ by means of complex coefficient $-J=\left|J\right|e^{i(\Theta +\pi )}$. This scaling presumes stretching/compression of the eigenvalue by factor |J| and rotation of the spectrum by angle Θ+π, see Figure 3.

Noteworthy, due to the assumption (15) for the eigenvalues, we formally obtain $\omega_{A}=\omega_{C}$, which implies

(28) $$\rho_{AC}\left(\omega_{A},\omega_{C}\right)=\rho_{A}\left(\omega_{A}\right)\delta \left(\omega_{C}-\omega_{A}\right).$$

**Figure 4:**
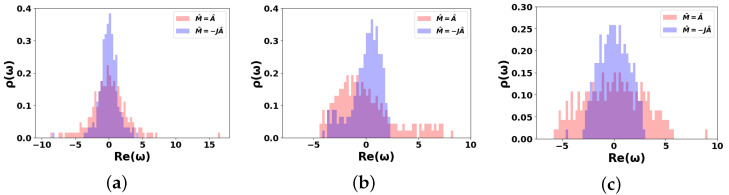
Spectral density ρ(ω) vs. real part of eigenfrequency Re(ω) for (**a**) scale-free graph, (**b**) WS graph with β=0.1, and (**c**) WS graph with β=0.9, see Figure 2a,c,e, respectively. $J=J_{R}-iJ_{I}$, where $J_{R}=J_{I}=0.5$, θ=π.

Substituting the (28) into the (13) and performing integration over the 2D complex plane, we find scaling equation

(29) $$\rho_{M}\left(\omega \right)=\frac{1}{{\left|J\right|}^{2}}\rho_{A}\left(\frac{\omega }{-J}\right),$$

where $\rho_{A}\left(\ldots \right)$ is the spectral density of the symmetric adjacency matrix $\hat{A}$. Figure 4 shows the spectral density $\rho \left(\omega \right)\equiv \rho_{M}\left(\omega \right)$ as a function of Re(ω) for the DIS networks discussed in this study (see also Figure 2). Firstly, let us focus on the red histograms in Figure 4, which correspond to spectral density $\rho_{A}\left(\omega_{A}\right)$ for scale-free and WS graphs, respectively.

To analyze the plots in Figure 4, it is useful to recall the definition of the spectral density, which characterizes the distribution of eigenvalues of the adjacency matrix and looks like (cf. [78])

(30) $$\rho \left(\omega \right)=\frac{1}{N}\sum_{n=1}^{N}\delta \left(\omega -\omega_{n}\right).$$

In particular, panels (b) and (c) of Figure 4 illustrate how the spectral density of WS graphs evolves as the rewiring probability β increases from 0.1 to 0.9, signaling a transition toward randomness. For sufficiently dense random graphs in limit N→∞, Equation (30) leads to the Wigner semicircle law upon averaging ρ(ω) over the ensemble of graph implementations, cf. [78,79]. For example, the bulk part of the red histogram in Figure 4c follows the Wigner semicircle law, which is characteristic of random graphs and is approached as β→1 in the WS model.

However, this approximation becomes invalid at low rewiring probabilities, i.e., for β close to zero, where the WS graph resembles a regular lattice. This is evident in Figure 4b, which shows numerous sharp peaks for the WS graph at β=0.1. In this regime, although some smoothing of the spectrum occurs, it remains largely quasi-discrete. Side peaks and flat-top structures are the features typical of periodic systems with small perturbations. Noteworthy, the kernel density estimation (KDE) can be employed to approximate the spectral density using a superposition of Gaussians (see, e.g., the blue histogram in Figure 4b), cf. [80].

In contrast, the adjacency matrix of the scale-free graph is sufficiently sparse that the Wigner semicircle law does not apply. Instead, the spectral density typically features a pronounced peak near ω=0 and a long positive tail, as seen in the red histogram in Figure 4a. For large ω, the tails of the spectral density for scale-free networks decay as ${\left|\omega \right|}^{-2\nu +1}$, assuming a degree exponent 2<ν<3 (cf. [34,36]).

Let us now turn to the renormalized spectral density, illustrated by the blue histograms in Figure 4. As evident from the plots, when |J|<1, the spectral density $\rho_{A}\left(\omega_{A}\right)$ of the original adjacency matrix is broader than that of the renormalized matrix ρ(ω). The negative sign in Equation (17) causes the spectral densities of the $\hat{M}$ matrix to be inverted relative to the vertical axis Re(ω)=0, shifting them into the negative Re(ω) region.

This inversion is clearly seen in Figure 4, where isolated peaks (corresponding to the PE and bulk components in Figure 4b) are mirrored. This is also consistent with Figure 3b. Notably, Equation (29) implies that the renormalization procedure preserves the scaling behavior of spectral density tails in the case of scale-free networks. Thus, we conclude that the renormalized spectral density retains its seminal features, and the analysis methods described above remain applicable.

### 4.2. Dis Network in the Presence of Inhomogeneous Coupling with the Reservoir

We now consider the case of inhomogeneous coupling between the DIS network and the reservoir, described by matrix $\hat{C}$ specified in (1), see also Figure 2.

Noteworthy, matrices $\hat{A}$ and $\hat{C}$ do not commute now, and (12) is not valid. In this case, Equation (18) takes form ($J_{I,eff}\equiv \cos\theta J_{I}\neq 0$):

(31) $$\omega_{n}E_{i}^{\left(n\right)}+J_{R}\sum_{j=1}^{N}A_{ij}E_{j}^{\left(n\right)}+iJ_{I,eff}\sum_{j=1}^{N}C_{ij}E_{j}^{\left(n\right)}=0,\;\;\;\;\;i=1,\ldots ,N.$$

**Figure 5:**
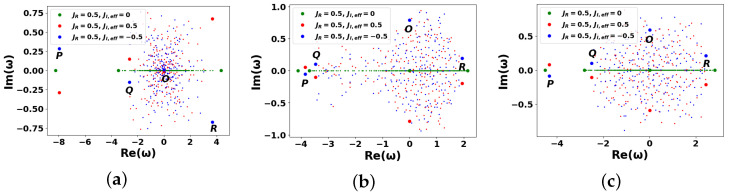
The same as in Figure 3, but for $C_{ij}$ specified in Equation (1). Nonzero weights $C_{ij}$ are random numbers normally distributed with mean value ${\bar{C}}_{ij}=0$ and variance σ=0.5. See more details in the text.

The numerical solutions of Equation (31) with ${\bar{C}}_{ij}=0$ and σ=0.5 are shown in Figure 5. As observed, the resulting spectra appear nearly chaotic, with a notable exception of cosθ=0, where the influence of inhomogeneous coupling $C_{ij}$ is effectively suppressed, see Figure 5a. This behavior arises because coupling weights $C_{ij}$ are randomly distributed and include both positive and negative values. In this regime, the DIS network exhibits behavior reminiscent of a *spin-glass-like* system, cf. [81,82]. Notably, according to the theory of collective opinion formation, negative weights $C_{ij}<0$ indicate the presence of opinions that are antagonistic towards the target community A (we assume that all matrix elements of A are non-negative), cf. [20].

Despite apparent randomness, PEs can still be identified in Figure 5a, which shows partial spectral structuring that can be attributed to the presence of hubs in the scale-free DIS network. Central eigenvalue $\omega_{O}≃0$ (point *O*) is clearly visible in the scale-free case (Figure 5a) but not for the WS graphs (see Figure 5b,c). On the other hand, the second and third eigenvalues (*Q* and *R*) possess no statistical significance under this regime and should not be interpreted structurally.

It is important to emphasize the statistically distinct behavior observed in the WS networks for different rewiring probabilities: β=0.1 (Figure 5b) and β=0.9 (Figure 5c). In the latter case, the spectrum appears completely random. This reflects the fact that the “regular” (ordered) component of the WS graph diminishes as β increases. At β≃1, the WS network exhibits the characteristics of a fully random graph. Consequently, Figure 5 provides clear evidence of a breakdown in the RG behavior observed in Figure 3, across all DIS network types considered.

**Figure 6:**
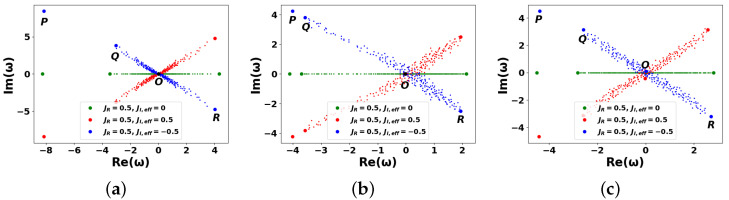
The same as in Figure 5, but for ${\bar{C}}_{ij}=1$. See more details in the text.

The situation changes significantly when coupling weights $C_{ij}$ are drawn from a normal distribution with non-zero mean ${\bar{C}}_{ij}\neq 0$. In Figure 6, we consider ${\bar{C}}_{ij}=1$. Under these conditions, the spectral properties of both the scale-free and WS networks shown in Figure 6 closely resemble, on average, their counterparts in Figure 3. The overall shape and alignment of the spectra are largely restored, indicating that a finite mean in the coupling distribution stabilizes coherent spectral features. In other words, dependencies in Figure 6 demonstrate smearing of the curves in Figure 3. Analytically, this can be explained as follows. We can assume that the eigenvalues of the $\hat{M}$ matrix (see (10)) consist of the real part, Re(ω), and the imaginary one, Im(ω), fluctuating as a normal random variable. This means that each eigenvalue of initial matrix $\hat{A}$ is “smeared” along the vertical (imaginary) axis Im(ω) with dispersion $J_{I,eff}^{2}\sigma^{2}$. As a result, spectral density $\rho_{M}\left(\omega \right)$ can be established as a convolution of spectrum $\hat{A}$ with a Gaussian along the Im(ω) axis in form

(32) ρM(ω)≈1|JR|ρARe(ω)−JR·12πJI,eff2σ2exp−(Im(ω)+JI,eff)22JI,eff2σ2,

where we suppose that ${\bar{C}}_{ij}=1$. Equation (32) characterizes a 2D spectral density in the complex plane. Along direction Re(ω), we obtain the spectral density scaled by the real factor $-J_{R}$, while in the Im(ω) direction, the density undergoes Gaussian broadening characterized by standard deviation $J_{I,eff}\sigma$ and mean displacement $-J_{I,eff}{\bar{C}}_{ij}=-J_{I,eff}$.

### 4.3. Spectral Entropy

Spectral entropy *S* is a useful tool to investigate the degree of disorder in spectral characteristics of various graphs, cf. [83,84,85,86]. We define *S* as follows

(33) $$S=-\sum_{n=1}^{N}p_{n}\log\left(p_{n}\right),$$

where

(34) $$p_{n}=\frac{|\omega_{n}|}{\sum_{m=1}^{N}\left|\omega_{m}\right|}$$

specifies the normalized absolute eigenvalues distribution for the $\hat{M}$ matrix, see (9); log(x) denotes the natural logarithm.

**Figure 7:**
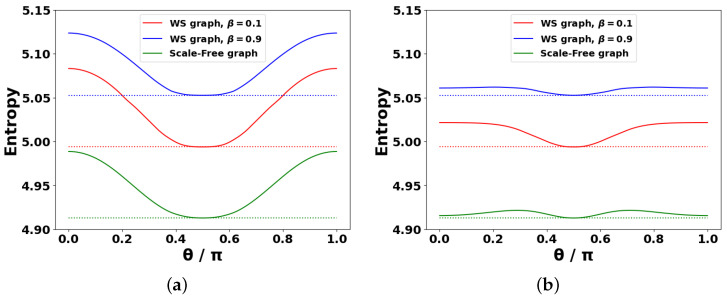
Spectral entropy *S* vs. parameter θ for (**a**) ${\bar{C}}_{ij}=0$, and (**b**) ${\bar{C}}_{ij}=1$, which are inherent to scale-free and WS DIS networks; σ=0.5. The bold solid curves correspond to $J_{R}=J_{I}=0.5$; the dotted lines correspond to $J_{R}=0.5$, $J_{I}=0$.

Figure 7 presents the dependence of spectral entropy on parameter θ, which characterizes the coupling between the DIS network and the external reservoir. Figure 7a relates to the spectral entropy that characterizes spin glass-like limit at ${\bar{C}}_{ij}=0$ (see Figure 5), while Figure 7b specifies *S* for ${\bar{C}}_{ij}=1$ (see Figure 6). As a result, a comparison between Figure 7a and Figure 7b reveals that more structured (ordered) interactions with the reservoir for a given graph topology lead to the reduction of spectral entropy.

As shown in Figure 7, the spectral entropy of the scale-free network (the green curves) is consistently lower than that of the WS networks (the red and blue curves). This reflects the greater spectral order inherent in scale-free structures, owing to the presence of hubs; cf. Figure 5a versus Figure 5b,c.

Moreover, the red solid curves lie below the blue ones, indicating that the spectral entropy of WS networks increases with rewiring probability β. This is attributed to the progressive disordering of the network topology as β grows. All solid curves in Figure 7 correspond to the case $J_{R}=J_{I}=0.5$, which leads to the following form of the $\hat{M}$ matrix: $\hat{M}=-\frac{1}{2}\left(\hat{A}+i\cos\theta \hat{C}\right)$. As a result, the maximal values of *S* in Figure 7a obtained at θ=0, θ=π, when $\hat{M}$ represents a balanced mixture of matrices $\hat{A}$ and $\hat{C}$ responsible for coherent and incoherent processes, respectively. Conversely, at θ=π/2, the coupling to the external reservoir vanishes, and $\hat{M}=-\frac{1}{2}\hat{A}$. In this purely coherent limit, all the solid curves in Figure 7 exhibit their minimum spectral entropy, reflecting the reduced spectral complexity of the system. This minimum is indicated by the green lines in Figure 3. The minima of spectral entropy are marked in Figure 7 with the dotted lines.

## 5. Phase Properties

To better understand the spectral properties of the DIS network shown in Figure 3, Figure 5 and Figure 6, we examined the phase distribution at characteristic points *P*, *Q*, *O*, and *R*. In this regard, it is instructive to specify an additional average local field ${\bar{E}}_{C,i}^{\left(n\right)}$

(35) $${\bar{E}}_{C,i}^{\left(n\right)}=\sum_{j=1}^{N}C_{ij}E_{j}^{\left(n\right)}=\left|{\bar{E}}_{C,i}^{\left(n\right)}\right|e^{i\chi_{C,i}^{\left(n\right)}},$$

where $\chi_{C,i}^{\left(n\right)}$ is the phase of the local average field associated with the connectivity matrix $\hat{C}$. Equations (19) and (35) allow us to rewrite (31) as

(36) $$e^{i\phi_{i}^{\left(n\right)}}\left(\omega_{n}|E_{i}^{\left(n\right)}|+J_{R}|{\bar{E}}_{A,i}^{\left(n\right)}|e^{i\Phi_{A,i}^{\left(n\right)}}+|J_{I,eff}\left|\right|{\bar{E}}_{C,i}^{\left(n\right)}|e^{i\Phi_{C,i}^{\left(n\right)}}\right)=0.$$

In Equation (36), we made definitions

(37) $$\Phi_{A,i}^{\left(n\right)}=\chi_{A,i}^{\left(n\right)}-\phi_{i}^{\left(n\right)},\;\;\;\;\;\Phi_{C,i}^{\left(n\right)}=\chi_{C,i}^{\left(n\right)}-\phi_{i}^{\left(n\right)}+\frac{\pi }{2}+\theta_{I},$$

where $\phi_{i}^{\left(n\right)}$ is the phase of $E_{i}^{\left(n\right)}$ ($E_{i}^{\left(n\right)}=\left|E_{i}^{\left(n\right)}\right|e^{i\phi_{i}^{\left(n\right)}}$), $J_{I,eff}=\left|J_{I,eff}\right|e^{i\theta_{I}}$.

The information field interference pattern at given node *i* is determined by the contributions of phase differences $\Phi_{A,i}^{\left(n\right)}$, $\Phi_{C,i}^{\left(n\right)}$, and $\Phi_{i}^{\left(n\right)}=\Phi_{C,i}^{\left(n\right)}-\Phi_{A,i}^{\left(n\right)}$, respectively. The phase $\Phi_{A,i}^{\left(n\right)}$ describes the influence of neighboring nodes on the *i*-th AIA–NIA pair, while $\Phi_{C,i}^{\left(n\right)}$ characterizes the contribution from the reservoir.

**Figure 8:**
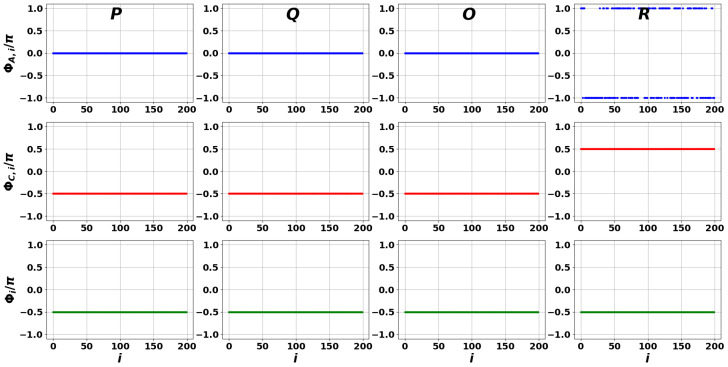
Phase distribution of the DIS scale-free network modes associated with points *P*, *Q*, *O*, and *R* shown in Figure 3a. The first, second, and third rows correspond to phase differences $\Phi_{A,i}^{\left(n\right)}$, $\Phi_{C,i}^{\left(n\right)}$, and $\Phi_{i}^{\left(n\right)}=\Phi_{C,i}^{\left(n\right)}-\Phi_{A,i}^{\left(n\right)}$, respectively.

**Figure 9:**
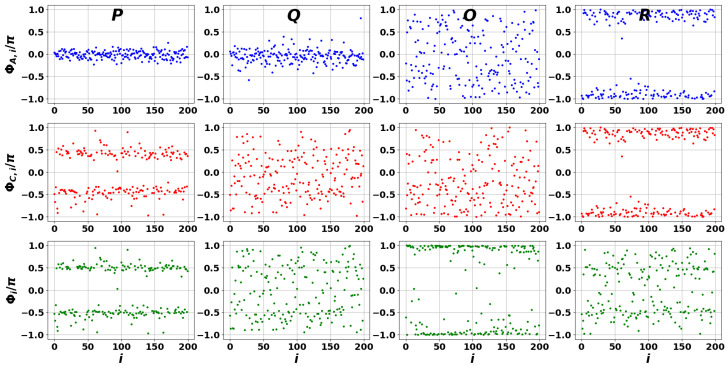
The same as in Figure 8 but for DIS scale-free network modes associated with Figure 5a.

**Figure 10:**
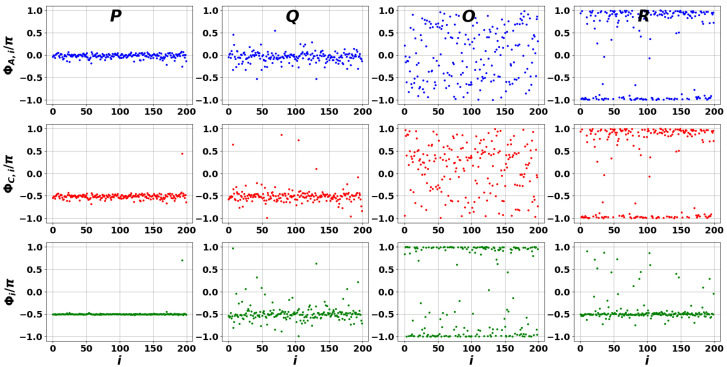
The same as in Figure 8 but for DIS scale-free network modes associated with Figure 6a.

Figure 8, Figure 9 and Figure 10 illustrate local interference effects between closely connected nodes, determined by phases $\Phi_{A,i}^{\left(n\right)}$, $\Phi_{C,i}^{\left(n\right)}$, and $\Phi_{i}^{\left(n\right)}$ for the case of a scale-free DIS network, cf. Figure 2a. In particular, Figure 8 clearly demonstrates a phase synchronization phenomenon. The universal behavior of the phases supports the RG conditions given by Equation (20). Notably, this effect is observed for all the points selected across the spectrum shown in Figure 3.

When the reservoir has specific properties determined by matrix $\hat{C}$, the local interference pattern is destroyed, as the phase distributions become smeared (see Figure 9 and Figure 10). In particular, the phase differences exhibit strong inhomogeneity at the selected *P*, *Q*, *O*, and *R* points of the spectra in Figure 5 and Figure 6, which account for the reservoir influence. Interestingly, some plots in Figure 10 display small phase fluctuations, which may be interpreted as partial synchronizability, cf. Figure 8 and Figure 9.

Our numerical simulations for the WS networks, performed in the same framework, revealed similar phase relationships to those shown in Figure 8, Figure 9 and Figure 10. This suggests that under strong environmental influence (increasing σ) DIS agents lose synchronization and mutual coherence abilities in their opinion formation. However, this conclusion does not hold for the Perron eigenfrequency.

**Figure 11:**
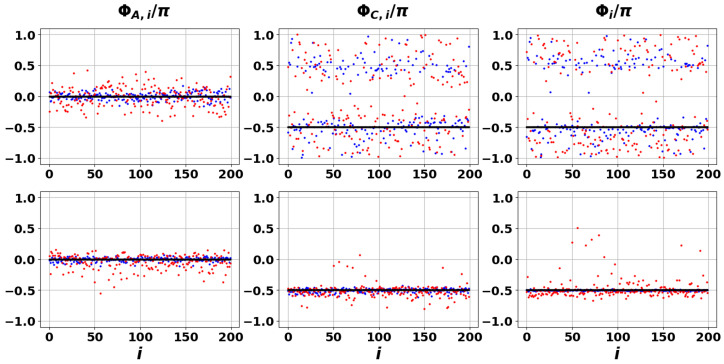
Phase distributions for the PEs, specified in Figure 3, Figure 5 and Figure 6 as *P*, respectively. The black dots correspond to the case $C_{ij}=A_{ij}$, see Figure 3, the blue dots are relevant to the case σ=0.5, see Figure 5 and Figure 6, the red dots correspond to the case σ=1.5. The first row corresponds to the case ${\bar{C}}_{ij}=0$, see Figure 5, the second row—to the case ${\bar{C}}_{ij}=1$, see Figure 6. The phases $\Phi_{A,i}$, $\Phi_{C,i}$, and $\Phi_{i}$ in columns are specified in Equation (37).

Figure 11 presents the dependencies of phases $\Phi_{A,i}^{\left(n\right)}$, $\Phi_{C,i}^{\left(n\right)}$, and $\Phi_{i}^{\left(n\right)}$ for the Perron eigenfrequencies across Figure 3, Figure 5 and Figure 6. The black lines indicate phase synchronization and correspond to the case in which the external reservoir is specified by the matrix $\hat{C}=\hat{A}$ (see Equation (15)), representing the RG limit. More intriguing, however, is the behavior of the blue and red points, which display the dependence of phases $\Phi_{A,i}^{\left(n\right)}$, $\Phi_{C,i}^{\left(n\right)}$, and $\Phi_{i}^{\left(n\right)}$ on σ for matrix $\hat{C}$ specified in Equation (1).

The lower panel of Figure 11 clearly shows that the examined phases exhibit only a weak dependence on increasing σ. This finding is of practical significance: it indicates that in the PE limit, the opinions of AIA–NIA pairs within the DIS network remain synchronized and robust against external information reservoir influences. Note that this occurs despite the fact that matrices $\hat{A}$, $\hat{C}$ do not commute with each other. Thus, we can conclude that the PE can also be recognized as a robust coherent state, cf. [87]. In contrast, for frequencies approaching central point *O*, the variations in phase distributions $\Phi_{A,i}^{\left(n\right)}$, $\Phi_{C,i}^{\left(n\right)}$, and $\Phi_{i}^{\left(n\right)}$ increase significantly, as seen in Figure 9 and Figure 10.

## 6. Conclusions

This work has examined a distributed intelligent system composed of AIA–NIA pairs interconnected through a complex network. Within each node, AIAs and NIAs interact closely with one another as well as with agents in other nodes, contributing to the formation of collective opinions and decisions reflected in the spectral properties of the DIS network. We propose the QGSP approach to research information propagation processes in the DIS. This approach takes into account quantum-like coherence and interference phenomena that occur during the information field spreading within the networks. For definiteness, we have considered two representative network topologies: a scale-free graph and a Watts–Strogatz graph. A distinctive feature of the problem is the potential for DIS agents to interact with an external environment represented by a large informational reservoir. To model this interaction, we employed a quantum formalism based on the GKSL equation. Within the mean-field approximation, we derived a system of equations that describe the propagation of the information field in the DIS. The influence of the reservoir was analyzed in two limiting cases.

First, adjacency matrix $\hat{A}$ of the DIS commutes with matrix $\hat{C}$ that characterizes coupling with external (reservoir) agents. In this case, we examine the limit when $\hat{C}$ represents a linear (with respect to the adjacency matrix) filter that affects the target community of the agents. Notably, despite the use of the mean-field approximation, which neglects quantum correlations, the network structures under study exhibit quantum-like properties. In the commuting case, we proposed an RG-based approach involving simple affine scaling of both the adjacency matrix and its spectral density. We showed that, in this regime, the reservoir has minimal impact on the spectrum of the DIS, which is governed by matrix $\hat{M}$—a superposition of $\hat{A}$ and $\hat{C}$, and reflects the process of collective opinion formation.

Second, we considered the limit when the influence matrix of reservoir $\hat{C}$ does not commute with $\hat{A}$; $\hat{C}$ possesses different weights from $\hat{A}$. In this case, the behavior of the eigenfrequencies becomes substantially richer, incorporating fluctuations in their imaginary parts arising from the normally distributed elements of the reservoir matrix $\hat{C}$. In this limit, the spectrum of $\hat{M}$ transitions to a quasi-ordered or fully disordered state. The latter occurs when the reservoir influence matrix $\hat{C}$ contains negative elements that are responsible for antagonistic information content in relation to the target community. Since we neglected the agents’ own beliefs, they can be strongly influenced by external information in this case.

Our results were further supported by an analysis of the spectral entropy and phase characteristics of $\hat{M}$. In particular, entropy specifies the disordered $\hat{M}$ matrix spectrum, taking into account both the coherent adjacency matrix and the incoherent influence matrix of reservoir $\hat{C}$ for the graphs under consideration. Importantly, the entropy reaches its maximum values at θ=0, θ=π corresponding to the strongest impact of the reservoir on the agent system. In contrast, at θ=π/2, when the reservoir is effectively absent, the entropy is minimized. It can also be seen from the behavior of spectral entropy in Figure 7 that the spectral characteristics of matrix $\hat{M}$ for a scale-free graph are more ordered than for a WS graph (the green curves occupy the lowest positions in Figure 7).

The phase of information field analysis accounts for the influence of both the information local mean fields generated by the DIS agents themselves and the external reservoir on an arbitrary *i*-th agent. We found that under the RG criterion, full synchronization of all phase parameters occurs, indicating coherence between the opinions of the *i*-th agent, its neighbors, and the reservoir. Conversely, in the non-commuting case, the reservoir induces disorder and desynchronization among the opinions of different DIS agents. This disorder intensifies near the central frequency region Re(ω)=0, while the Perron frequency mode remains robust to external perturbations and is essentially unaffected by disorder parameter σ. These spectral properties of DIS networks may prove valuable for the analysis and design of multi-agent systems, particularly those incorporating agents based on LLMs and related architectures.

Finally, we briefly outline several promising directions for future research where the proposed theoretical framework may offer valuable insights. These directions stem from the approximations and limitations considered in the present study. One important avenue involves extending the model to sociodynamic processes and opinion formation with a focus on consensus-building in the DIS, which was not the primary focus of this work. In this context, it would be important to incorporate agent self-confidence features and to consider the case of a symmetric reservoir influence matrix. This limit corresponds to scenarios in which reservoir agents receive no feedback from the target community. In addition, it would be useful to analyze cases where the order of the polynomial filter *K* exceeds one, implying that the reservoir influence on each agent is more diffusely distributed across the network. Another promising direction is the simulation of the proposed model using LLM-based agents, assigning them DIS-like profiles tailored to specific domains such as economics or social sciences. These application-specific studies are highly relevant and currently in demand, and they require dedicated problem formulations adapted to the respective contexts.

## Data Availability

The original contributions presented in this study are included in the article. Further inquiries can be directed to the corresponding author.
